# Revealing the potential of non-transplanted donor organs: a comparative analysis in the Eurotransplant region

**DOI:** 10.3389/ti.2026.16516

**Published:** 2026-08-03

**Authors:** A. Kofler, F. J. Krendl, L. Prommegger, J. Hofmann, A. Weissenbacher, B. Cardini, A. T. Meszaros, T. Resch, G. Brandacher, T. Hautz, R. Oberhuber, E. de Buijzer, S. Eschertzhuber, S. Schneeberger

**Affiliations:** 1 Department of Visceral, Transplant and Thoracic Surgery, Center for Operative Medicine, Medical University of Innsbruck, Innsbruck, Austria; 2 Eurotransplant International Foundation, Leiden, Netherlands; 3 Department of Anesthesia and Intensive Care Medicine, Regional Hospital, Hall in Tirol, Austria

**Keywords:** biomedical research, eurotransplant, legal framework, non-transplanted organs, organ donation

## Abstract

Post-mortem organ donation is essential for modern transplantation medicine, yet a substantial proportion of recovered donor organs are not transplanted. These organs, although clinically declined, hold significant and untapped value for biomedical research and technological innovation. Eurotransplant reports and United States national data were reviewed to quantify transplanted and not used organs and to assess temporal trends. During the study period, 100,260 organs were reported in Eurotransplant, of which 32,242 (32.16%) were not transplanted. Of all reported organs, 16.05% were accepted for transplantation but not used, representing a stable and predictable number of organs suitable for secondary research use. A review of international, European, and national legislation demonstrated that, in many Eurotransplant countries, existing laws focus almost exclusively on transplantation and provide limited guidance on the research use of non-transplanted organs, with the Netherlands, Slovenia, Luxembourg, and partially Belgium as notable exceptions. Emerging technologies highlight the scientific and clinical relevance of these organs. Establishing legal and ethical pathways for their secondary use could reduce organ wastage, support innovation, and improve transplantation outcomes. Engagement with the Austrian Federal Ministry of Health, informed by the findings of this work, contributed to an amendment of the Austrian Organ Transplantation Act.

## Graphical Abstract

**Figure:**
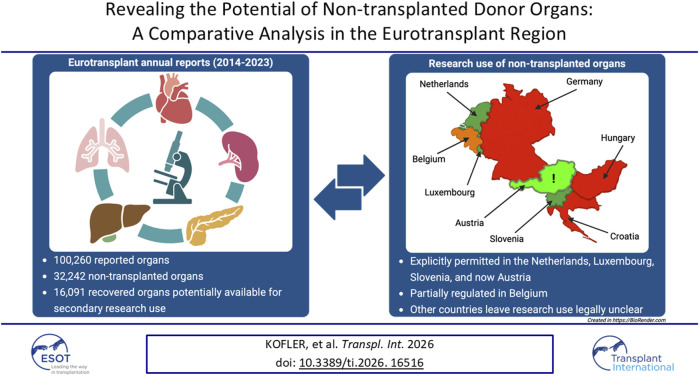
Created in BioRender.

## Introduction

Initiated in 1967 by Professor Jon J. van Rood, the Eurotransplant International Foundation (Eurotransplant) is a non-profit organization coordinating the allocation of post-mortem donor organs in 8 European countries. Member countries of Eurotransplant are Austria, Belgium, Croatia, Germany, Hungary, Luxembourg, Netherlands and Slovenia. Eurotransplant plays a central role in the just and efficient distribution of organs such as kidneys, livers, hearts, lungs, pancreases and intestines.^1^ Allocation is based on medical and ethical criteria [1, 2]. The most important criteria for organ allocation are blood group, donor size and age, tissue characteristics (HLA groups especially for kidneys), clinical urgency, waiting time, and regional availability (depending on organ type).^2^

Three consent models exist for deceased organ donation. In Eurotransplant, Germany adopted the informed consent (opt-in) model, where the donor or their designated surrogate must explicitly confirm the donor’s willingness to donate before organ recovery. All other Eurotransplant countries apply an opt-out model based on presumed consent. Individuals are considered organ donors upon death unless they have registered an objection.^3^ The third is the explicit authorization model, adopted in the United States, which requires explicit authorization from the donor emphasizing individual autonomy.^4^ Each system reflects a different ethical and legal position on balancing personal choice with the need to maximize organ availability for transplantation.

The decision-making of organ suitability is guided by donor and recipient factors, transportation logistics, hospital and center policies, time of day, and, most importantly, the subjective assessment of the surgeons at the transplant centers. Notably, non-utilization rates increase during weekends and nighttime hours, highlighting the impact of human factors such as workload and decision fatigue [3, 4]. Hence, organ non-utilization is driven by subjective factors such as institutional risk aversion, labeling biases, and inconsistent interpretation of imperfect clinical data [5]. To reduce unnecessary organ non-utilization, it is crucial to establish more objective and standardized criteria.

Despite the efforts towards organ donation in the Eurotransplant region, the shortage of donor organs (1,424 waiting-list-deaths in 2024;^5^ 140 million population^6^) remains the key limiting factor in organ transplantation. Novel medical tools for organ preservation combined with the development of new technologies for assessment and treatment of donor organs [6, 7] enable for data-based organ quality assessment and higher organ utilization rates.

Normothermic machine perfusion (NMP) enables safe transplantation of high-risk donor livers that would otherwise not be utilized [8]. Normothermic regional perfusion (NRP) is used in donation after circulatory death (DCD) and has demonstrated higher utilization rates and better outcomes [9]. Machine perfusion has enabled functional assessment and recovery of marginal liver [10–18], kidney [19], pancreas and lung [20, 21] grafts. In pancreas transplantation, *ex vivo* NMP has been applied in an experimental setting and select centers to assess function in declined grafts [22, 23]. *Ex situ* organ perfusion platforms also serve to test perfusion solutions and additives under controlled conditions [24–26]. Broader use of perfusion technologies may help meet transplant demand [8, 27]. However, the challenge lies in deciphering the significance of this information to determine its most effective and efficient use in organ transplantation.

The accelerating pace of technological advancement has dramatically expanded the capacity to gather, analyze, and interpret data. Combined with artificial intelligence, machine learning, and biomedical imaging, this fuels the prospect of a more robust, data-driven and fair allocation system.

To bridge the widening gap between data acquisition and biological insight, it is imperative to obtain a greater number and diversity of human organs for research. The study of organs across different ages, ethnicities, disease states, and environmental backgrounds, may reveal meaningful biological signals with predictive value towards the outcome after transplantation. Therefore, expanding access to human organs, that otherwise would not be used is essential for fully realizing the potential of contemporary and future biomedical technologies.

The aim of this manuscript is to assess the current state of research on human organs deemed unsuitable for transplantation and to examine the legal framework and practical application across Eurotransplant. It highlights the need for improved policies and handling of these organs to expand research opportunities and increase organ availability.

## Materials and methods

### Data sources

Annual Eurotransplant reports from 2014 to 2023 were used as primary data source.^7^

To provide a comparison with organ utilization in the United States, statistics from 2014 to 2023 were obtained from the Annual Data Reports of the US Organ Procurement and Transplantation Network and the Scientific Registry of Transplant Recipients (OPTN/SRTR).^8^

The analysis includes only deceased-donor organs.

Organs are counted individually; lungs and kidneys separately, livers as whole organs (with splits counted separately) and hearts, pancreases, and intestines per organ.

### Study variables

Annual data from 2014 to 2023 were obtained from Eurotransplant for kidney, liver, heart, lung, and pancreas transplantation. The total number of reported donor organs and their subsequent allocation outcomes were extracted. Reported organs were categorized according to their progression through the allocation process into mutually exclusive categories defined by Eurotransplant: (i) reported, (ii) not offered, (iii) offered but not accepted, (iv) accepted but not transplanted, and (v) transplanted.

For analytical purposes additional descriptive groupings were used. All organs that were not transplanted were collectively referred to as non-transplanted organs, regardless of the reason or stage which they exited the transplantation destination. The term “discarded” was avoided, in line with consensus in the field [28].

Within the non-transplanted group, organs categorized as accepted by a transplant center but eventually not transplanted were specifically referred to as “recovered organs” for the purpose of this analysis. Original Eurotransplant outcome categories were retained for reporting and figures. This category corresponds conceptually to “recovered organs” in the United States reporting system, representing organs recovered for transplantation but not ultimately transplanted.

Organ-specific utilization patterns were evaluated by comparing the proportion of transplanted organs across organ types.

For the United States comparator analysis, aggregated annual data from 2014 to 2023 were extracted from OPTN/SRTR annual reports. These data included counts of reported donor organs, transplanted organs, non-transplanted organs, reported categories of non-use, and organs used for scientific or technical purposes.

### Utilization rate calculation

Organ-specific utilization rates were calculated by dividing the number of transplanted organs with the total number of reported organs for each organ type and expressing the result as a percentage.

### Statistical analysis

Descriptive statistics were calculated for each annual variable. Normality was assessed using the Shapiro–Wilk test. To evaluate temporal trends over the ten-year observation period, linear regression analyses with year as a continuous predictor were performed for each outcome variable.

### Cross-country comparison

To contextualize Eurotransplant findings, we compared reported organ numbers and utilization outcomes with United States OPTN/SRTR statistics over the same period (2014–2023). Per-million-population (pmp) values were interpreted using Eurostat and United States Census estimates to enable direct comparison between regions and over time.

### Legal and policy assessment

A structured document review was conducted of relevant international, European, and national legal instruments in all Eurotransplant member states. For each country, we identified the consent model and examined whether the respective transplantation laws explicitly addressed the research use of non-transplanted organs and especially recovered organs, whether additional consent beyond donation consent was required for research use, and whether national operational guidance or legislation referred to Eurotransplant policies governing the secondary use of declined organs. Findings were narratively synthesized by country.

## Results

### Organ utilization efficiency in eurotransplant

**FIGURE 1:**
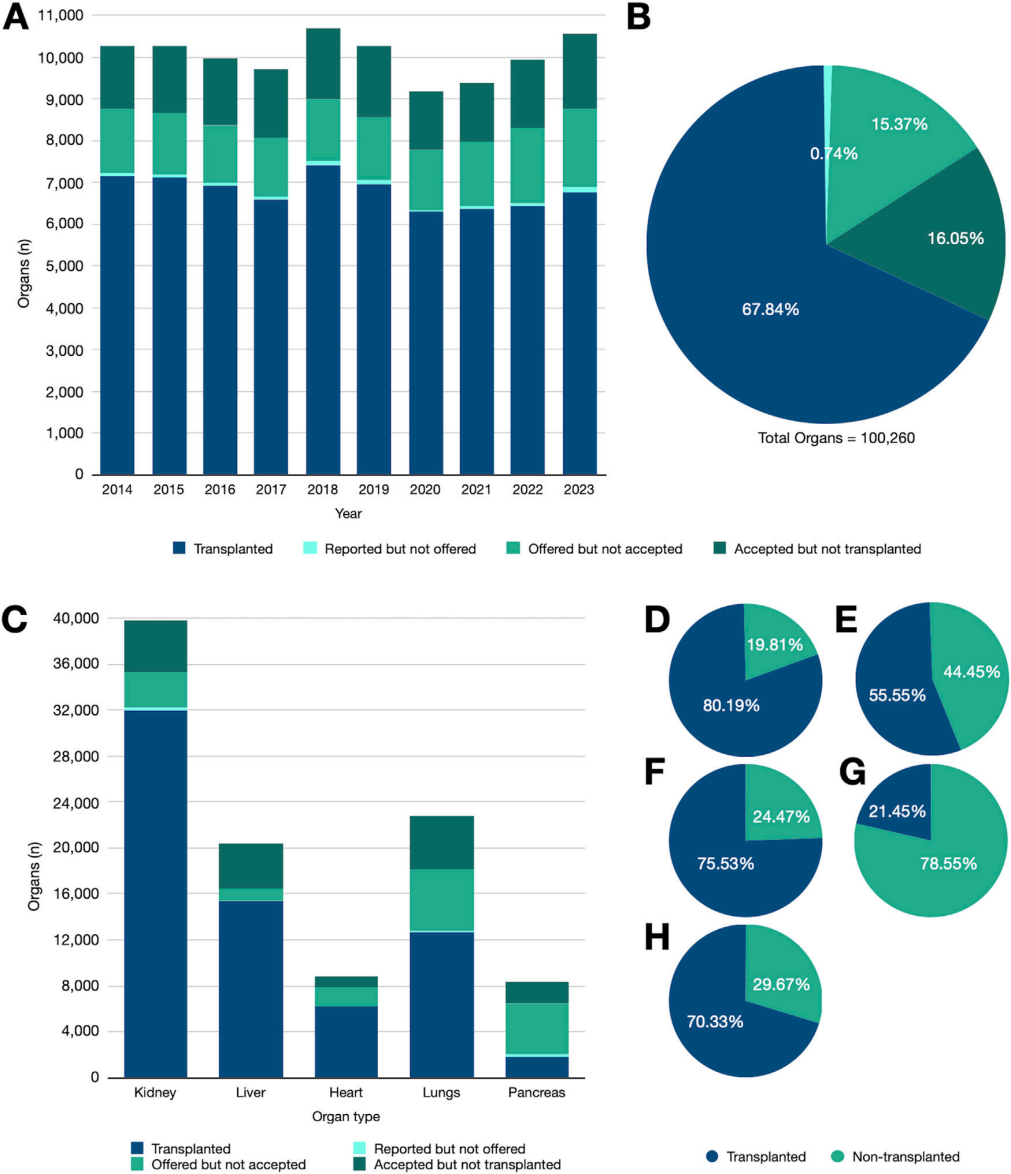
Fate of all reported donor organs in the Eurotransplant region from 2014 to 2023. **(A)** Yearly distribution of reported donor organs according to their destination (not offered, not accepted, not transplanted, and transplanted), illustrating temporal trends in organ utilization over the ten-year period. **(B)** Cumulative distribution of all reported donor organs during the study period, categorized by final destination, providing an overall overview of organ utilization and non-utilization. **(C)** Cumulative number of reported organs stratified by organ type. **(D–H)** Percentage distribution of transplanted versus non-transplanted organs by organ type: **(D)** kidney, **(E)** lungs, **(F)** liver, **(G)** pancreas, and **(H)** heart.

A total of 100,260 organs (kidney, liver, heart, lungs, pancreas) were reported for transplantation between 2014 and 2023 (as shown in Table 1).^7^ Of these, 32,242 (32.16%) were ultimately not suitable for transplantation. From the total number of non-transplanted organs, three subgroups can be identified: 740 (0.74%) organs were never offered for transplantation, 15,411 (15.37%) organs were offered but not accepted by any transplant center, 16,091 (16.05%) organs were initially accepted for transplantation, but the procedure was ultimately not carried out due to various reasons such as unexpected pathologies, poor organ function, histological findings, and others.^7^ Also, organ utilization rates varied by organ. Kidneys had a utilization rate of 80.19%, while pancreas utilization was 21.45% (Table 2; Figure 1). The number of reported, transplanted, and not transplanted organs over the ten-year observation period is depicted in Figure 1.

**TABLE 1:** Number of reported and non-transplanted organs from 2014 to 2023 in the Eurotransplant region.

Year	Organs reported	Reported but not offered	Offered but not accepted	Accepted but not transplanted	Non-transplanted organs (total)
​	n	n (%)	n (%)	n (%)	n (%)
2014	10,270	89 (0.87)	1,522 (14.82)	1,523 (14.83)	3,134 (30.52)
2015	10,271	68 (0.66)	1,458 (14.20)	1,615 (15.72)	3,141 (30.58)
2016	9,965	42 (0.42)	1,385 (13.90)	1,600 (16.06)	3,027 (30.38)
2017	9,695	52 (0.54)	1,422 (14.67)	1,624 (16.75)	3,098 (31.95)
2018	10,705	76 (0.71)	1,483 (13.85)	1,724 (16.10)	3,283 (30.67)
2019	10,259	80 (0.78)	1,522 (14.84)	1,693 (16.50)	3,295 (32.12)
2020	9,192	57 (0.62)	1,415 (15.39)	1,429 (15.55)	2,901 (31.56)
2021	9,382	82 (0.87)	1,532 (16.33)	1,417 (15.10)	3,031 (32.31)
2022	9,949	77 (0.77)	1,790 (17.99)	1,655 (16.63)	3,522 (35.40)
2023	10,572	117 (1.11)	1,882 (17.80)	1,811 (17.13)	3,810 (36.04)
Total	100,260	740 (0.74)	15,411 (15.37)	16,091 (16.05)	32,242 (32.16)

Non-transplanted organs are categorized as not offered, offered but not accepted, and accepted but not transplanted. The table also shows the percentage of each category relative to the total number of reported organs per year.

Minor discrepancies may occur due to rounding of percentage values.

**TABLE 2:** Reported and non-transplanted organs by organ type in the Eurotransplant region from 2014 to 2023.

Organ	Organs reported	Reported but not offered	Offered but not accepted	Accepted but not transplanted	Non-transplanted organs (total)
​	n	n (%)	n (%)	n (%)	n (%)
Kidney	39,837	283 (0.71)	3,021 (7.58)	4,587 (11.51)	7,891 (19.81)
Liver	20,399	41 (0.20)	1,018 (4.99)	3,933 (19.28)	4,992 (24.47)
Heart	8,790	43 (0.49)	1,629 (18.53)	936 (10.65)	2,608 (29.67)
Lungs	22,827	117 (0.51)	5,293 (23.19)	4,737 (20.75)	10,147 (44.45)
Pancreas	8,407	256 (3.05)	4,450 (52.93)	1,898 (22.58)	6,604 (78.55)

Nonutilized organs are classified as not offered, offered but not accepted, or accepted but not transplanted. The table additionally presents the percentage of each category in relation to the annual total number of reported organs.

The median annual number of transplanted organs was 6,850 (IQR = 703; n = 10 years; β = −76.4 organs/year; R2 = 0.37; p = 0.062), indicating no statistically significant trend over time. The category “reported but not offered” had an annual median of 76.5 organs (IQR = 25; n = 10 years; β = +3.2 organs/year; R^2^ = 0.21; p = 0.177), likewise showing no significant temporal change. For “offered but not accepted”, the median was 1,502.5 (IQR = 110; n = 10 years; β = +38.3 organs/year; R^2^ = 0.50; p = 0.023), representing the only category with statistically significant upward trend over the study period. The “accepted but not transplanted” category showed a median annual count of 1,619.5 (IQR = 170; n = 10 years; β = +8.1 organs/year; R^2^ = 0.04; p = 0.585), indicating a stable volume. The total number of reported organs had a median of 10,112 (IQR = 576; n = 10 years; β = −26.7 organs/year; R^2^ = 0.03; p = 0.649). The total annual number of non-transplanted organs had a median of 3,137.5 (IQR = 264; n = 10 years; β = +49.6 organs/year; R^2^ = 0.31; p = 0.093).

### Donor utilization efficiency

**FIGURE 2:**
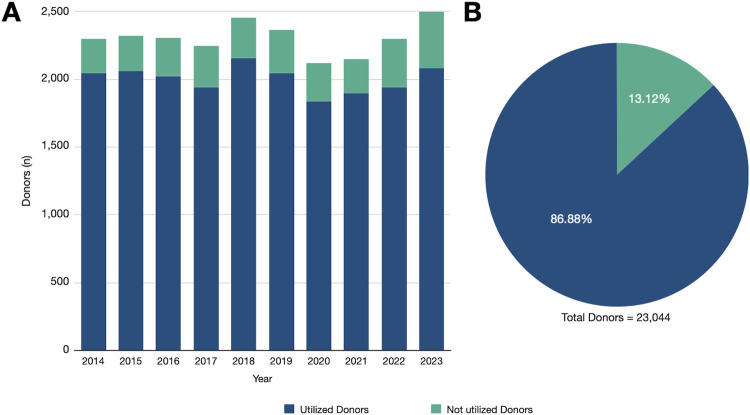
Fate of all reported donors in the Eurotransplant region from 2014 to 2023. **(A)** Yearly distribution of reported donors according to their utilization status, illustrating temporal trends and changes in donor utilization rates over the ten-year study period. **(B)** Cumulative distribution of all reported donors from 2014 to 2023, summarizing overall utilization and highlighting the proportion of donors who were not ultimately utilized for transplantation despite being reported.

The number of reported, utilized, and not utilized donors over the ten-year observation period is depicted in Figure 2. The total number of organ donors reported from 2014 to 2023 in the Eurotransplant region was 23,044 (as shown in Table 3). Of these, 3,024 (13.12%) donors were not considered suitable for donation.^7^ The annual number of reported donors ranged from 2,119 to 2,499 across the ten-year study period (mean 2,304; SD = 118.8; n = 10 years).

**TABLE 3:** Number of reported and utilized Donors from 2014 to 2023 in the Eurotransplant region.

Year	Reported donors	Utilized donors	Not utilized donors
​	n	n (%)	n (%)
2014	2,299	2,041 (88.78)	258 (11.22)
2015	2,317	2,063 (89.04)	254 (10.96)
2016	2,306	2,021 (87.64)	285 (12.36)
2017	2,246	1,942 (86.46)	304 (13.54)
2018	2,455	2,159 (87.94)	296 (12.06)
2019	2,362	2,042 (86.45)	320 (13.55)
2020	2,119	1,837 (86.69)	282 (13.31)
2021	2,146	1,898 (88.44)	248 (11.56)
2022	2,295	1,938 (84.44)	357 (15.56)
2023	2,499	2,079 (83.19)	420 (16.81)
Total	23,044	20,020 (86.88)	3,024 (13.12)

The table also shows the percentage of each category relative to the total number of reported Donors per year.

The annual number of not utilized donors ranged from 248 to 420 (mean 302.4; SD = 52.87; n = 10 years). Linear regression analysis showed a significant upward trend in not utilized donors over time (β = +11.8 donors/year; R^2^ = 0.459, p = 0.031).

Eurotransplant data specifies only limited information regarding why these donors were deemed unsuitable for transplantation. In total, 3,024 donors, corresponding to potentially 24,192 organs, were not utilized.

### Comparison of organ utilization efficiency with the United States

**FIGURE 3:**
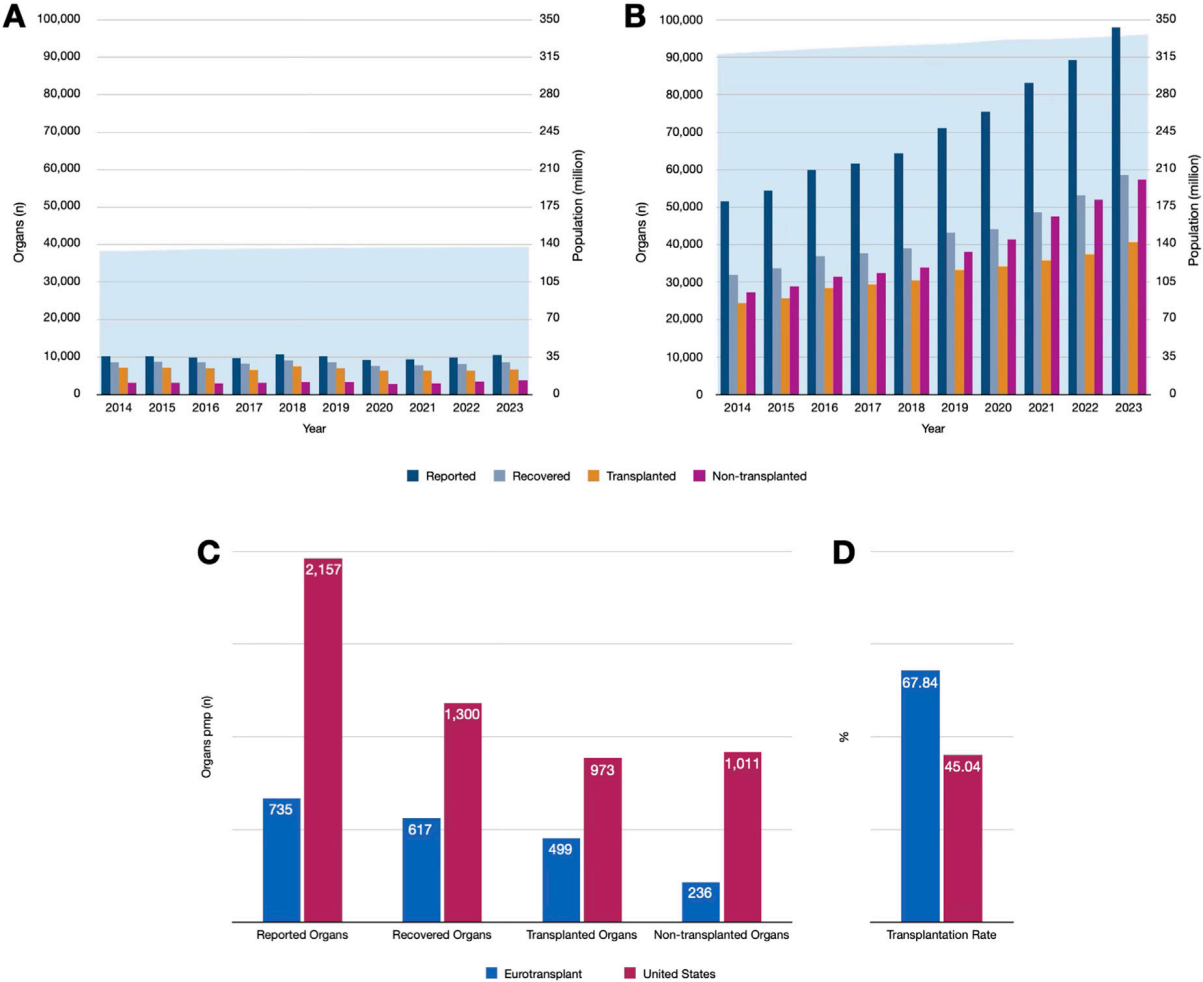
Comparison of organ transplantation efficiency in Eurotransplant and the United States from 2014 to 2023. **(A)** Organ activity in Eurotransplant (reported, recovered, transplanted, not transplanted); primary axis: absolute organ numbers; secondary axis: per million population (pmp), shown as blue shaded background. **(B)** Organ activity in the United States with the same categories and axis structure as in **(A)**. **(C)** Head-to-head comparison of organ activity per million population (pmp) between Eurotransplant and the United States across all categories. **(D)** Direct comparison of organ transplantation efficiency (%) between Eurotransplant and the United States (transplanted relative to reported organs).

**FIGURE 4:**
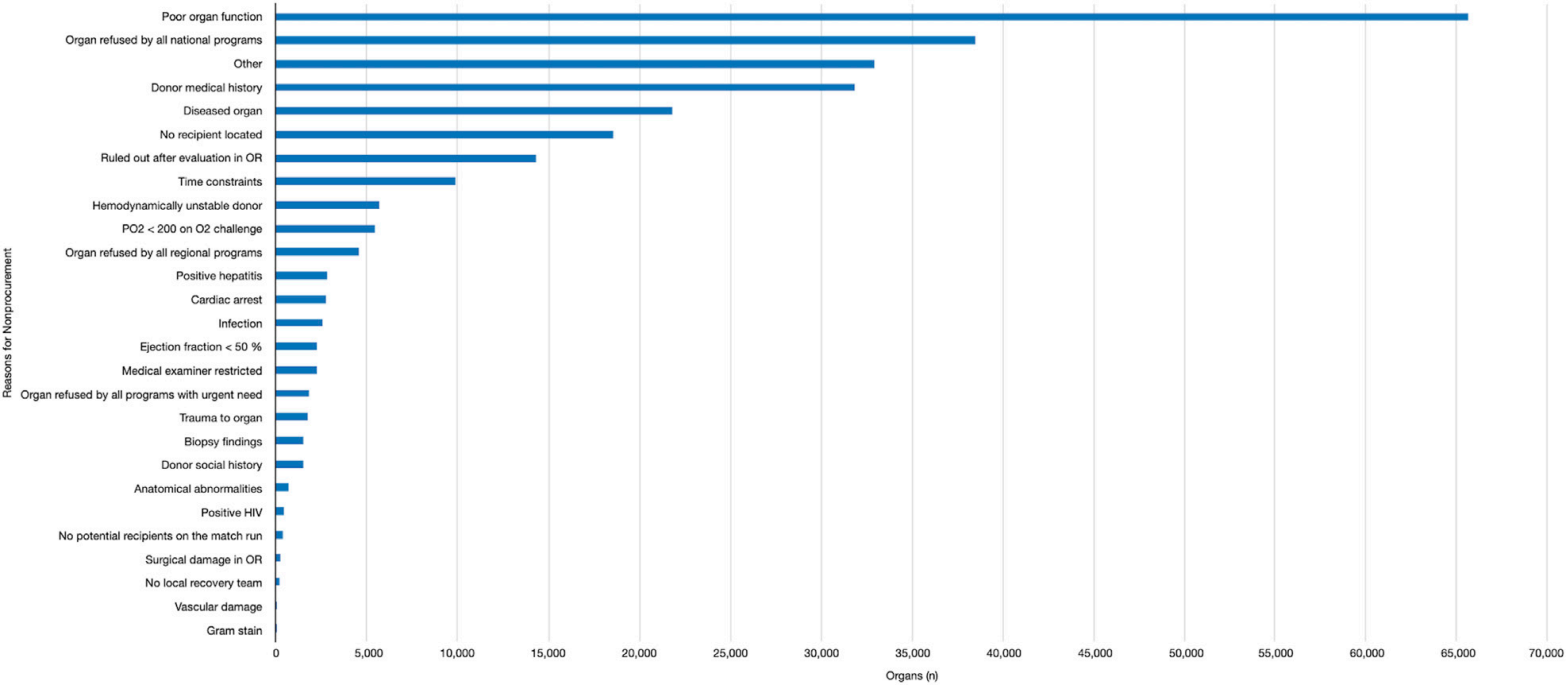
Reasons for non-procurement of reported donor organs in the United States from 2014 to 2023.

The number of reported donor organs differed considerably between the Eurotransplant region and the United States (735 pmp vs. 2,157 pmp respectively; Figure 3). From 2014 to 2023 a total of 709,099 organs were reported in the United States, of which 319,410 (45.04%) organs were transplanted and 332,072 (46.83%) were not. This number of non-transplanted organs is composed of organs, for which consent for donation was not requested, consent was not obtained, and organs which were not recovered due to, e.g., presumed poor organ function, organ disease, donor medical history, time constraints, infection, trauma, anatomical or vascular damage, biopsy findings, etc. (Figure 4). From the 49,435 (6.97% of reported) organs used for science, a proportion (75.69%) was recovered with the immediate intention to use the organs for research while the remaining organs (24.31%) were recovered with the intent of transplantation but later repurposed for scientific use. Organs recovered for technical evaluations, heart valve procurement, islet cell isolation, hepatocyte extraction, etc., included 8,182 (1.15% of reported) organs.

The current reality is that the number of transplanted organs pmp differs substantially between Eurotransplant and the United States (499 pmp vs. 973 pmp). When Germany is excluded from the assessment, the ET transplantation rate is 657 pmp, indicating a regional effect in the ET data. The number of organs used for research are displayed in Table 4.

**TABLE 4:** Organ donation and utilization in Eurotransplant and the United States from 2014 to 2023.

2014–2023	Eurotransplant n (%, pmp)	United States n (%, pmp)
Reported organs	100,260 (100, 735)	709,099 (100, 2,157)
Non-transplanted organs	32,242 (32.16, 236)	332,072 (46.83, 1,011)
Transplanted organs	68,018 (67.84, 499)	319,410 (45.04, 973)
Organs used for research	*not available*	49,435 (6.97, 150)
Organs used for others*	*not available*	8,182 (1.15, 25)
Recovered organs**	84,109 (83.89, 617)	427,029 (60.22, 1300)

* Other includes technical evaluations, heart valve procurement, islet cell isolation, hepatocyte extraction, etc.

** It is certain that organs within Eurotransplant were also used for research. However, Eurotransplant does not report separate categories for such secondary uses and only distinguishes between transplanted and not transplanted organs. Consequently, the Eurotransplant category “non-transplanted” may include both absolute non-utilization and scientific utilization, but this distinction cannot be quantified. The chosen approach represents the most methodologically consistent strategy given the available data, while acknowledging that differences in reporting granularity between the systems may introduce a degree of residual uncertainty.

Comparison of organ donation data between Eurotransplant and the United States, presented as absolute numbers, percentages, and per million population (pmp, figures are rounded), including reported, non-transplanted and transplanted organs, as well as organs assigned for research and other purposes.

### Reasons for non-procurement

There are several reasons, why donor organs may not be used, both during the initial assessment stage and after provisional acceptance. Common factors include extended cold ischemia time, organ damage arising either from the retrieval process or from donor-related trauma, and anatomical abnormalities. Other reasons are poor organ function, the donor’s medical history (e.g., comorbidities, infections, or malignancies) and donor age, logistical challenges such as transport, inability to locate a suitable recipient, or a positive crossmatch with the recipient. Center-specific and conservative acceptance criteria, recipient health status, and the need for a better immunological match may also contribute to an organ not being used for transplantation (Figure 4) [5, 29, 30].

### The legal framework

**FIGURE 5:**
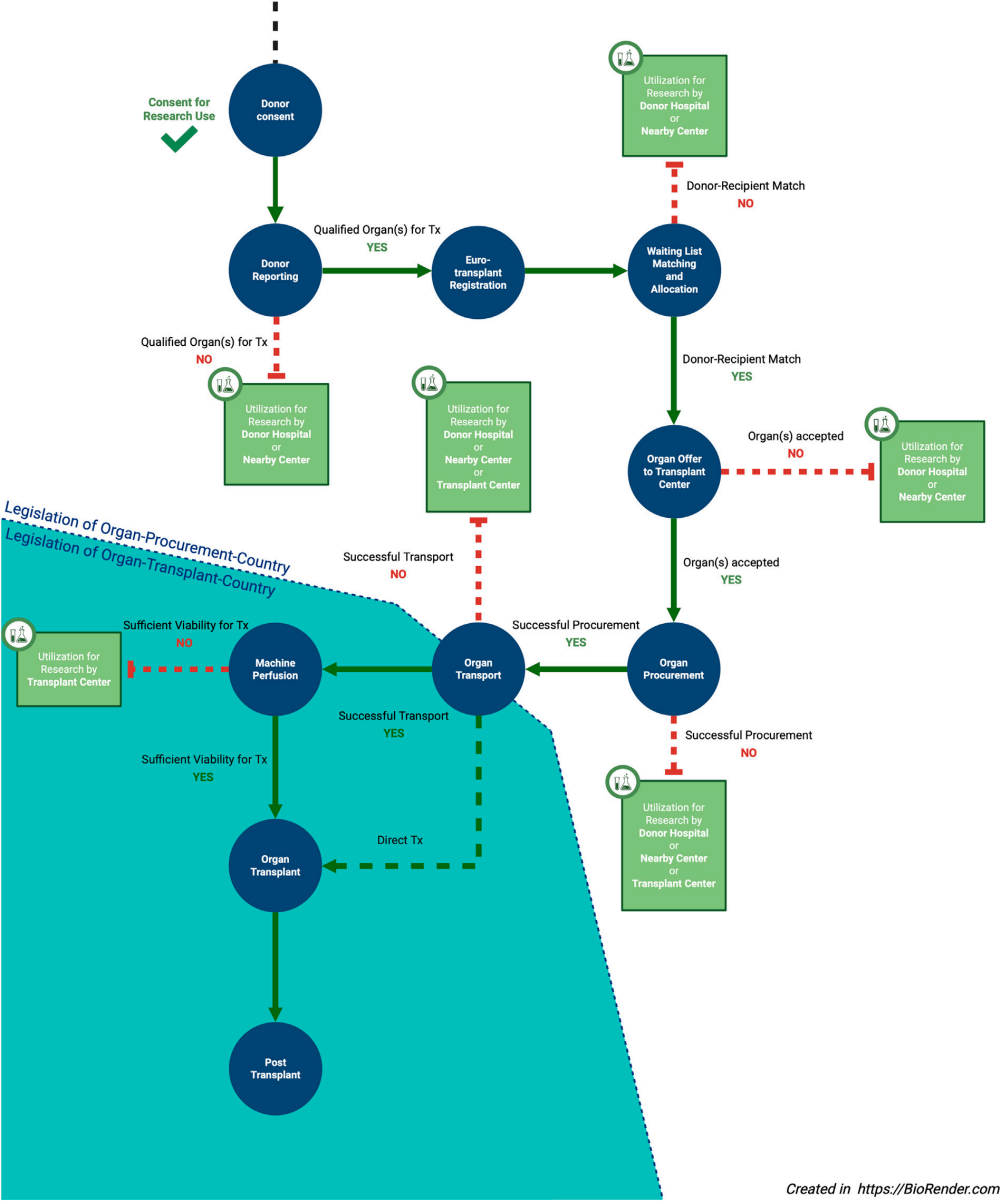
Organ pathway from donation to transplantation. Schematic overview of the organ pathway from donation consent to transplantation, illustrating key stages and decision points where organ abandonment can occur. The figure indicates the stakeholders involved at each step and identifies who may potentially access the organ for research purposes if it is not used for transplantation and after consultation with Eurotransplant. It also marks the point where jurisdiction and applicable legislation may change if the organ is transported to another country. Created in BioRender. Kofler, A. (2026) https://BioRender.com/5scwhix.

Key international frameworks guiding ethical organ donation and transplantation include United Nations General Assembly Resolutions 71/322, 73/189, 75/195, and 77/236 [31], the Declaration of Istanbul of 2008 [32], the World Health Organization Guiding Principles on Human Cell, Tissue and Organ Transplantation of 2010 [33], and the World Health Assembly Resolution 62.22 of 2009 [34]. Together, they promote transparency, prohibit organ trafficking and transplant tourism, emphasize voluntary and informed consent, and call for equitable access and international cooperation in transplantation practices. The pathway of a donor organ from donation consent to transplantation crosses several institutional and, at times, national boundaries, and at each stage distinct legal instruments determine who may hold, assess, or ultimately use the organ, as summarized in Figure 5.

In Europe, key legal instruments include the European Treaty Series No. 005 [35], No. 050 [36], No. 164 [37], and No. 186 [38], as well as Council of Europe Treaty Series No. 197 [39], and No. 216 [40]. They are all issued under the framework of the Council of Europe, an intergovernmental organization founded in 1949 to promote human rights, democracy, and the rule of law across its member states.^9^ These documents collectively establish ethical and legal standards for organ and tissue donation, ensure informed consent and donor protection, regulate the cross-border exchange of human substances, and explicitly prohibit trafficking in organs and humans for the purpose of organ removal.

At European Union level, the main legal instruments are Directive 2010/53/EU [41] (complementing Directive 2004/23/EC) and Implementing Directive 2012/25/EU [42]. Directive 2010/53/EU sets minimum quality and safety standards for organs intended for transplantation, whereas Implementing Directive 2012/25/EU regulates cross-border exchange, traceability, and communication between member states.

Directive 2010/53/EU serves as the central legal basis for the regulations of the member states of the European Union and is intended to ensure high medical quality and the protection of organ donors and recipients. The harmonization of regulations at European level is intended to ensure that organs can be transplanted under comparable and safe conditions within the EU, regardless of their country of origin. The directive thus forms the basis for national legislation on organ donation and transplantation and contributes to improving the availability and quality of donor organs [43].

As a directive, it is intended to be transposed into national law by EU member states, where each member state determines the form and the methods for the incorporation.^10^ In some, existing laws were amended to align with the directive, whereas in others, new legislation was enacted to ensure compliance.

Directive 2010/53/EU accentuates the removal of organs for transplantation and the research on those organs, but it remains silent on the fate of organs that are unsuitable for transplantation from the outset or are deemed unfit during retrieval, transport, or prior to implantation. On the other hand, Implementing Directive 2012/25/EU regulating transparency and traceability in cross-border organ exchanges by mandating that Member States notify each other either of the recipient’s identity (via a national code) or, if the organ was not transplanted, of its final use. Importantly, the directive does not define or limit what constitutes “final use” in cases where an organ is not transplanted. Conceptually, this lack of specification leaves the organs deemed unsuitable for transplantation, available to be repurposed for alternative uses, including scientific research, provided that this is in compliance with national laws and ethical guidelines.

For the Eurotransplant member states, the organization’s regulatory instruments serve as a supporting reference. These include the Ethical Charter and the Eurotransplant Manual, which provide binding guidance on operational and ethical standards. The prerequisite for any use of organs beyond transplantation is the decline for transplantation. This includes the use of the organ for isolation of cells or tissues, or for research purposes. The allowance for any such use of organs is regulated by the national legislation. If no allowance exists, the organ must either be cremated, disposed of by another approved method, or returned to the donor country. Alternatively, the organ may remain with the donor.^11^

### Eurotransplant countries’ transplant policies

The following sections summarize the relevant legal provisions for each Eurotransplant member state. A comparative overview is provided in Table 5.

**TABLE 5:** Overview of Eurotransplant member countries by donation system, legal framework for research, practical use of non-transplanted organs for research, and availability of body donation programs.

Country	Donation system	Explicit statutory approval for organ-based research	Body donation program
Austria	Opt-out	Yes*	Yes
Belgium	Opt-out	No	Yes
Croatia	Opt-out	No	Yes
Germany	Opt-in	No	Yes
Hungary	Opt-out	No	Yes
Luxembourg	Opt-out	Yes	No
Netherlands	Opt-out	Yes	Yes
Slovenia	Opt-out	Yes	Yes

This table summarizes key characteristics of Eurotransplant member countries, including whether they operate under opt-in or opt-out organ donation systems, their legal provisions for using non-transplanted organs in scientific research, the extent to which such research is actively conducted, and the presence of formal body donation systems for medical education or research.

* An amendment to the Austrian Organ Transplantation Act, passed unanimously by the National Council on 20 May 2026, now explicitly permits the scientific use of procured organs that could not be transplanted.

#### Austria

In Austria, the Organ Transplantation Act (*Organtransplantationsgesetz*) (OTPG) of 2012 governs the removal and transplantation of organs and implements Directive 2010/53/EU. The OTPG does not apply to the use of organs for scientific purposes. Therefore, the use of removed organs for research is not regulated by the OTPG [44].

Austria follows an opt-out system for organ donation: anyone who has not objected during their lifetime is considered a donor. However, this presumed consent applies only to transplantation [45].

Research on non-transplanted organs is not explicitly regulated within the donation process. If an organ is considered unsuitable for transplantation, a separate determination must be made as to whether it can be used for research. In practice, the deceased had given prior consent for their organs to be used for scientific purposes if not transplantable. Without such consent, unsuitable organs are typically disposed of through regulated medical waste procedures [46].

Austrian law explicitly refers to Eurotransplant in the context of vigilance and surveillance. Eurotransplant provisions therefore apply within this framework.^12^

Organ donation is centrally coordinated. The Austrian health authorities and the Austrian National Public Health Institute, maintain registers, and organ allocation is organized by Eurotransplant.^13^

There are also provisions for anatomical donation (body donation for scientific purposes), but these must be considered separately.^14^ A non-transplanted organ could, in principle, be treated as part of such a donation, provided appropriate consent exists. However, there is no specific regulation that comprehensively governs research use of non-transplanted or already recovered organs.

Subsequent to the completion of this analysis, engagement with the Austrian Federal Ministry of Health contributed to an amendment of the OTPG, passed unanimously by the National Council on 20 May 2026, which explicitly clarifies the legal permissibility of science use of procured organs that could not be transplanted, closing the regulatory gap described above.^15^

#### Belgium

In Belgium, the opt-out system applies to post-mortem organ retrieval [47]. The use of recovered organs for research purposes is not directly regulated by the Law on the Retrieval and Transplantation of Organs (*Loi du 13 juin 1986 sur le prélèvement et la transplantation d’organes)*, which was revised in 2012 to align with EU legislation [48]. This law states that recovered organs can be used for research purposes only if they are intended to be transplanted into the human body. Research involving organs that have been deemed unsuitable for transplantation is permitted but not necessarily covered by consent to organ donation. Instead, a different legal framework might apply: the Law on the Procurement and Use of Human Bodily Material for Human Medical Applications or Scientific Research (*Loi du 19 décembre 2008 relative à l’obtention et à l’utilisation de matériel corporel humain*) [49]. According to this legislation, explanted but non-transplanted organs could be classified as human bodily material and may be used for research purposes. In practice, explicit consent is required.

Transplant coordinators manage the transplantation process in collaboration with Eurotransplant, ensuring effective coordination between donor hospitals and transplant centers.^16^

In Belgium, as in many other European countries, it is also possible to donate the body after death for medical training or research. However, the Federal Public Service for Health distinguishes this from organ donation, noting that organs are only removed for transplantation purposes not for research.^17^

#### Croatia

Croatia has adopted the opt-out system since 1988 [50, 51]. The Law on the removal and transplantation of human body parts for medical purposes (*Zakon o uzimanju i presađivanju dijelova ljudskog tijela u svrhu liječenja*) permits post-mortem organ retrieval provided that the individual did not object during their lifetime [52]. This legislation is exclusively focused on transplantation. It does not explicitly regulate the research use of organs that have been deemed unsuitable for transplantation. Official oversight lies with the Ministry of Health and the National Transplantation Organization [50].

In exceptional cases, with approval from the Croatian Medical Chamber’s ethical committee, the body of a deceased person who did not live with family and did not object in writing may be used for medical training, provided no next of kin object in writing.^18^

In Croatia, individuals can also choose to donate their bodies after death to support medical education and scientific research.^19^

#### Germany

The Transplantation Act *(Transplantationsgesetz)* (TPG) of 1997 exclusively governs the donation, removal, allocation, and transplantation of organs [53]. Research involving non-transplanted organs does not fall within the scope of the TPG.

The current legal framework regarding organ donation in Germany is known as the opt-in-related “informed consent” model. This means that organ donation is generally only possible if the potential donor gave consent during their lifetime, for example, by registering their decision in the national organ donor registry, or if the next of kin have given their approval [54]. The removal of organs is only permitted for the purpose of transplantation. This means that if an organ is removed but not transplanted, it may not automatically be used for research purposes. Without such specific consent, a non-transplanted organ must generally be properly disposed of [55].

There is no specific legal regulation that explicitly permits or prohibits the use of postmortem-removed organs for research purposes. The organ donor card in Germany provides the option to include additional notes, such as giving consent for research.^20^

Oversight in Germany is regulated by the German Organ Procurement Organization and Eurotransplant.^21^

In Germany, individuals may also choose to donate their bodies posthumously for medical education and scientific research.^22^

#### Hungary

Hungary regulates organ and tissue donation under the Healthcare Act of 1997 *(1997 évi CLIV. törvény az egészségügyről)*, specifically the chapter on organ transplantation [56]. Additional provisions are contained in Ministerial Decree 18/1998 on Transplantation. According to this law, organ retrieval from deceased individuals is permitted in the absence of a documented objection, hence establishing an opt-out system [57]. However, this legally presumed consent applies exclusively to transplantation for therapeutic purposes. The use of recovered organs for other purposes, such as scientific research, is not included under this presumed consent. More, organs removed from the deceased for transplantation but not used shall be subjected to histopathological examination.^23^ Although the Hungarian Health Act does not mention the use of non-transplanted organs for research purposes, it does include provisions that allow for the transport of organs, including across national borders, for various purposes, one of which is scientific research.

Organ transplantation is centrally coordinated by the Organ Coordination Office of the Hungarian National Blood Transfusion Service.^24^

Hungary also offers a body donation program, enabling individuals to contribute their bodies after death to support medical education and scientific research.^25^

#### Luxembourg

Luxembourg enacted the Law on Organ Procurement *(Loi du 25 novembre 1982 relative au prélèvement d’organes)* as early as 1982 declaring all residents to be potential organ donors unless they have formally objected [58]. This law has since been amended to incorporate EU legislation. The opt-out system applies to organ retrieval for transplantation and scientific purposes, but the 1982 legislation does not explicitly address the possible use of non-transplanted organs for research. This core legislation has been amended notably by the Law of 25 June 2015 *(Loi du 25 juin 2015 modifiant la loi du 25 novembre 1982 réglant le prélèvement de substances d’origine humaine)*, to update or adapt provisions in line with the EU directive [59]. Additionally, there are several Grand-Ducal-Regulations covering further aspects of organ transplantation.

Organ allocation is coordinated at the national level under the authority of the Ministry of Health, with operational responsibilities managed by Luxembourg-Transplant. The allocation process is integrated into the broader international framework of Eurotransplant.^26^

It remains unclear whether a formal body donation program exists in Luxembourg, as no publicly available information or official framework has been identified.

#### Netherlands

The initial law governing organ donation in the Netherlands was the Law on Burial and Cremation *(Wet op de Lijkbezorging)*, adopted in 1986 [60]. This law included provisions allowing for post-mortem organ and tissue donation, provided the deceased had not objected during their lifetime. It established a system based on presumed consent. The 1986 law was later replaced by the Organ Donation Act *(Wet op de Orgaandonatie)* in 1996, due to growing complexity of transplantation practices, and last amended in 2020 [61].

Netherlands is the Eurotransplant member state with the most clearly defined legal framework that explicitly permits the use of declined donor organs for scientific research, stating that *“consent, […] (or lack of objection), is granted for the purpose of implantation, including scientific research aimed at implantation, if the organ after removal is found to be unsuitable for implantation”*. This means that if an individual does not object in the donor register, organs that are recovered but cannot be transplanted into a recipient may be used for scientific research aimed at advancing transplantation medicine. This use is legally permitted without requiring additional consent from the next of kin, unless the deceased explicitly stated during their lifetime that their organs must not be used for research purposes.^27^

The Ministry of Health, Welfare and Sport, the Donor Registry, and the Dutch Transplantation Foundation maintain continuous coordination with Eurotransplant to optimize the cross-border allocation, matching, and transplantation of donor organs throughout the participating countries.^28^

In the Netherlands, individuals can choose to donate their bodies after death to support medical research and education through an official body donation program.^29^

#### Slovenia

In Slovenia, the main legal document is the Act on Regulation, the Procurement and Transplantation of Human Body Parts for the Purposes of Medical Treatment of 2000 *(Zakon o odvzemu in presaditvi delov človeškega telesa zaradi zdravljenja)* [62]. Organs that have been recovered but are subsequently deemed unsuitable for transplantation fall outside the specific legal framework of this law. This is where the 2015 Regulation on the Traceability and Disposal of Human Organs Intended for Transplantation and on the National Identification Number *(Pravilnik o sledljivosti in uničenju človeških organov namenjenih za presaditev ter o nacionalni identifikacijski številki)* comes into effect [63]. If an organ is found unsuitable for transplantation, it may be recalled and offered to other EU countries. If no country accepts it, the law states that *“in the case of prior consent of the donor, [the organ can be] used for the purposes of medical education or scientific research”.*

Organ donation and allocation is centrally coordinated by the National Transplant Institute*,* which is responsible for reporting available organs to Eurotransplant and ensuring full compliance with applicable national and international regulation.^30^

Slovenia also has a body donation program that allows individuals to donate their bodies to medical education and research after death.^31^

### Laws for cross-border organ exchange

The applicable jurisdiction in the context of cross-border organ exchange within the EU primarily is likely to depend on the physical location of the organ at each stage of the process (Figure 5). The national law of the country where the organ is located governs the medical, ethical, and procedural aspects of organ procurement, testing, and storage. Once the organ crosses the border into another EU member state, it can be assumed that the legal framework of the recipient country becomes applicable, especially concerning transplantation procedures, patient safety standards, and documentation requirements. The national laws in this area are based on Directive 2010/53/EU, which sets minimum quality and safety standards for the donation, procurement, and transplantation of human organs across the EU. The Directive also emphasizes that health policy and transplantation systems remain under Member State competence. The principle is reinforced by Article 168 of the Treaty on the Functioning of the European Union, which requires EU health actions to respect Member State responsibility for healthcare systems. As a result, while the EU provides a harmonized framework for quality and safety, legal authority and jurisdiction in transplantation remain determined by national law. Jurisdiction therefore appears to be territorially anchored, with the national law of each state applying to activities carried out within its borders [41, 42, 64].

## Discussion

Post-mortem organ donation remains a cornerstone of modern transplantation medicine, enabling life-saving treatments and improving quality of life. The public and academic discourse primarily focuses on successfully transplanted organs. Less attention is paid to organs recovered but ultimately deemed unsuitable for transplantation. It is important to emphasize from the outset that transplantation must remain the goal and overarching priority for every recovered organ. Any framework enabling research use of non-transplanted organs should only apply after all efforts towards transplantation have been exhausted. In case an organ is deemed non-transplantable, research addressing innovations that may foster to make currently non-transplantable organs transplantable in the future should be given priority over other research interests.

Non-transplanted organs hold considerable scientific value that remains largely untapped. Between 2014 and 2023, 332,072 and 32,242 organs were recovered but not utilized in the US and Eurotransplant, respectively. These numbers illustrate the huge potential in this field. Interestingly, organ utilization rates vary also by organ type. Abdominal organs (kidney, liver, and pancreas) tolerate longer cold ischemia times than thoracic organs and show heterogeneous acceptance rates. In contrast, thoracic organs (heart and lungs) have higher immediate functional requirements and limited ischemic tolerance, leading to more conservative donor selection and lower utilization [65–69]. The introduction of machine perfusion (NMP, HOPE, NRP) has mitigated some of these limitations by enabling functional assessment and extending preservation times^32^ [9, 27].

A deceased donor organ may be unsuitable for transplantation yet transformative for research and development. Utilizing non-transplantable organs can support the development and validation of novel diagnostic tools, surgical techniques, and therapeutic approaches under conditions closely reflecting human physiology. Moreover, biomedical research often struggles to account for the variability in human populations, which limits the effectiveness of pre-clinical models. Studying human organs declined for transplantation offers a powerful alternative for assessment prior to clinical trials. Such organs allow for early assessment of viability and function. However, establishing the viability of such organs for transplantation remains inherently challenging. Unless the organ is ultimately transplanted and its function can be assessed *in vivo*, any conclusions remain speculative [27, 70]. While cell cultures and animal models remain valuable, their role should follow validation in human tissue. Once validated, animal models could then provide deeper mechanistic insights (or *vice versa*) [71]. Repurposing procured but not transplantable human organs for research directly aligns with 3R principle of Directive 2010/63/EU [72] by replacing less predictive animal models, reducing unnecessary animal use, and refining preclinical testing.

This manuscript covers three distinct categories of not transplantable organs: those deemed unsuitable before retrieval and therefore not even offered for transplantation, those offered for transplantation but not accepted by any transplant center, and those accepted for transplantation but excluded during the procurement or evaluation process and ultimately not used. Among these, particular attention should be given to organs that were initially accepted and procured but ultimately not transplanted (herein referred to as recovered organs). These organs represent a valuable resource because they have passed selection and logistical assessments and are already recovered. Therefore, future efforts in policy, ethics, and clinical practice should focus on this group, exploring safe and ethically sound ways to repurpose them for research, without compromising donor dignity or public trust. Utilizing organs that have already been recovered may also reduce dependence on animal testing. This is in line with broader ethical and scientific objectives.

Over the last 10 years, the number of non-transplanted organs within ET was stable. On average, 16.05% of all reported organs would qualify for potential secondary use under this framework. This consistency suggests a predictable source of organs for research. However, many Eurotransplant member countries lack a clear legal framework that governs the research use of organs not intended for transplantation. Current legislation is focused on transplantation, leaving little room for alternative, science-driven applications. This lack of regulation leads to uncertainty regarding research use. As a result, organs that could significantly advance medical knowledge are often disposed of.

What applies to non-transplanted organs also holds true for non-realized donors. The organs from these donors may hold considerable value. While the reasons for non-utilization of those donor organs are diverse and sometimes vague, the added value of using these organs for advancement and development in the field of transplantation and beyond needs to be considered. More, the conceptual link from unused organs to organ donors and further to whole-body donation for, e.g., anatomical education illustrates the close connection between these topics. At the same time, it reflects a broader societal attitude toward the idea of making one’s own body available for scientific purposes. This connection emphasizes not only the individual willingness to contribute to research but also society’s need to make use of such opportunities in order to advance medical knowledge and achieve improvements for the benefit of humanity.

The model of whole-body donation for research purposes is well established and widely accepted. Extending this framework to include individual organs that are not viable for transplantation appears ethically consistent. Such an extension would enhance scientific opportunities while respecting donor intent.

International comparisons highlight the feasibility of more inclusive policies. In the US research access to not transplantable donor organs is supported by both legal and institutional mechanisms, facilitating ethically sound and scientifically valuable work [73]. The number of reported donor organs differs considerably between the Eurotransplant region and the United States (735 pmp vs. 2,157 pmp respectively). Key factors influencing this discrepancy include the high donor rate resulting from the stronger incentivization system and the national guidance and coordination in the United States, the opioid crisis, as well as the low donor rate in Germany. Reasons why this system may capture a broader range of potential donors, including those whose organs are ultimately not used. While the incentive-based approach can be a strong driving force, it also exposes the system to scrutiny and demands strong objectivity in the decision-making process [74]. Hence, comparisons between reported donor numbers should be made with caution, as the underlying systems and reporting practices may differ. Anyway, these practices could serve as a point of reference for European countries seeking to modernize and refine their regulatory approaches.

An illustrative example from the United States is 34 Lives, a public benefit corporation dedicated to improving the utilization of viable donor kidneys at risk of not being utilized. 34 Lives employs advanced organ preservation strategies to sustain and, in some cases, restore kidney function. Preliminary data indicate that the initiative has led to higher transplantation rates, underscoring its potential to reduce organ wastage and improve outcomes for transplant recipients.^32^

Emerging technologies such as machine perfusion make it increasingly important to clarify access to non-transplanted human organs. In the liver, NMP has demonstrated feasibility for perfusion periods of up to 48 h, and even 7 days in some cases [75, 76], while maintaining key metabolic and structural functions. A human liver was perfused *ex vivo* for 3 days and subsequently transplanted, with the patient recovering normal liver function and quality of life without signs of rejection or bile duct injury after 1 year of follow-up [77]. Transcriptomic and metabolomic analyses during NMP have revealed activation of pro-survival and repair pathways in livers, along with effective autophagy and redox balance [78–80]. Composite viability criteria such as lactate clearance, pH, bile output, and vascular flow patterns have been successfully used to guide transplantation decisions [10, 12]. Long-term perfusion has also enabled metabolic interventions such as steatosis reduction [81, 82]. In addition, senolytic treatments applied during *ex vivo* liver perfusion have shown promise in preserving the regenerative capacity of the biliary system, by mitigating the detrimental effects of cellular senescence on cholangiocyte function and primary cilia integrity, which are crucial for biliary repair [83, 84]. In machine perfused human livers, adeno-associated virus (AAV) vectors were compared under neutralizing and non-neutralizing conditions, revealing distinct differences in transduction efficiency and antibody susceptibility [85]. Single-cell analyses further demonstrated that vector performance depends on liver condition and capsid choice [86]. Split-liver perfusion models enable direct intra-organ comparison of therapeutic interventions [87–89]. As a preclinical test, a liver from a patient with mitochondrial neurogastrointestinal encephalomyopathy was used to demonstrate AAV-mediated gene therapy in a human organ indicating feasibility of correcting the genetic defect [90].

In the kidney, 24- to 48-h normothermic perfusion with urine recirculation has shown stable flow and resistance, acid-base balance, and tissue morphology enabling functional assessment of organ condition [91–94]. Kidney NMP may eventually reduce non-transplantation rates and improve allocation [95, 96]. A study demonstrated that non-transplanted human kidneys can be preserved in a metabolically active and functionally stable state for up to 4 days using subnormothermic (25 °C) machine perfusion [97–99].

In the pancreas, NMP has been successfully applied in research settings to assess function in non-transplanted grafts. Stable perfusion, insulin secretion, and minimal histological injury was observed in preliminary studies [22, 23]. This approach remains investigational and is not currently part of routine clinical practice. Hypothermic perfusion also enables isolation of viable islets from DCD donor pancreases [100].

In lung transplantation, advances in machine perfusion have helped to preserve and treat organs. Techniques such as prone positioning may improve lower lobe function and reduce reperfusion injury [21]. Moreover, xenogeneic cross-circulation has demonstrated potential for the extracorporeal recovery of severely injured human lungs [20]. Some lungs initially deemed unsuitable were later found transplantable [21].

Beyond the immediate clinical use cases, *ex situ* organ perfusion facilitates decellularization of whole human livers and kidneys and generation of biological bioscaffolds [98, 99, 101]. In an attempt to generate bioengineered transplantable organs, these scaffolds have been reseeded with human cells. Delivery of multipotent adult progenitor cells during liver NMP resulted in transendothelial migration and secretion of immunomodulatory factors, demonstrating compatibility of cell therapy with perfusion platforms [102].

In conclusion, organs that have already been recovered but not transplanted should not be automatically excluded from further use. When guided by robust ethical standards and clear legal frameworks, their use in biomedical research represents an opportunity to advance science, possibly reduce animal testing, and ultimately benefit future patients. Achieving this will require public dialogue, political initiative, and a shift in perspective. Such an effort would also render organ donation more meaningful and, in the long term, more effective.

## Data Availability

The original contributions presented in the study are included in the article, further inquiries can be directed to the corresponding author.

## Footnotes

1 Eurotransplant International Foundation. Cooperating saves lives. https://www.eurotransplant.org/about-eurotransplant/cooperating-saves-lives/ [Accessed February 14, 2026].

2 Eurotransplant International Foundation. Eurotransplant Manual, Chapter 1: Introduction (2022). https://www.eurotransplant.org/wp-content/uploads/2022/02/H1-Introduction-July-28-2016.pdf [Accessed October 12, 2025].

3 Eurotransplant International Foundation. Legislation. https://www.eurotransplant.org/region/legislation/ [Accessed October 14, 2025].

4 Organ Procurement & Transplantation Network. Ethics of deceased organ donor recovery (2016). https://optn.transplant.hrsa.gov/professionals/by-topic/ethical-considerations/ethics-of-deceased-organ-donor-recovery/ [Accessed June 21, 2025].

5 Council of Europe. Newsletter Transplant 2024: International Figures on Organ, Tissue and Hematopoietic Stem Cell Donation and Transplantation Activities. Strasbourg: European Directorate for the Quality of Medicines & Healthcare (EDQM) (2024). https://www.edqm.eu/en/-/newsletter-transplant-2024-organ-donation-increases-globally [Accessed September 3, 2025].

6 Eurotransplant International Foundation. Annual Report (2023). https://cdn.sanity.io/files/ngz8tmzz/production/24445ce7c521641ca1cd28e24ff7ff2442576fcb.pdf [Accessed September 4, 2025].

7 Eurotransplant International Foundation. Annual Reports. https://www.eurotransplant.org/statistics/annual-report [Accessed August 28, 2024].

8 Organ Procurement & Transplantation Network. Annual Data Reports. https://srtr.transplant.hrsa.gov [Accessed September 7, 2024].

9 Council of Europe - Conseil de l'Europe. The Council of Europe at a glance (2023). https://www.coe.int/en/web/portal/the-council-of-europe-at-a-glance [Accessed March 5, 2025].

10 EUR-Lex Access to European Union Law. Summaries of EU legislation: Directive. https://eur-lex.europa.eu/legal-content/EN/TXT/?uri=LEGISSUM:directive [Accessed March 3, 2025].

11 Eurotransplant International Foundation. Annual Report (2014). https://cdn.sanity.io/files/ngz8tmzz/production/8b30d3687225237b1f9b73ca6f39828699d9c856.pdf [Accessed January 18, 2024].

12 Eurotransplant International Foundation. Eurotransplant Manual, Chapter 3: Allocation General (2022). https://www.eurotransplant.org/wp-content/uploads/2022/02/H3-Allocation-v-4.2-January-2022.pdf [Accessed October 12, 2025].

13 oesterreich.gv.at. Organisationsstruktur der Organtransplantation (2025). https://www.oesterreich.gv.at/de/themen/gesundheit/organtransplantation/1 [Accessed May 8, 2025].

14 Medizinische Universität Wien. Körperspenden an die Forschung. https://www.meduniwien.ac.at/web/klinik-gesundheit/ambulanzen-services/koerperspenden-an-die-forschung/ [Accessed May 8, 2025].

15 Parlament Österreich, Parlamentskorrespondenz Nr. 439 vom 20.05.2026, Nationalrat stärkt Schutz vor kommerziellem Organhandel. https://www.parlament.gv.at/aktuelles/pk/jahr_2026/pk0439 [Accessed June 21, 2026].

16 Health Food Chain Safety Environment. Transplant coordinators (2016). https://www.health.belgium.be/en/transplant-coordinators [Accessed April 8, 2025].

17 Santé publique. Don d’organes et don de corps à la science (2016). https://www.health.belgium.be/fr/sante/prenez-soin-de-vous/debut-et-fin-de-vie/don-dorganes/citoyens/don-dorganes-et-don-de-corps-la [Accessed April 8, 2025].

18 Government of the Republic of Croatia. Organ donation. https://gov.hr/en/organ-donation/782 [Accessed July 15, 2025].

19 Zavod za Anatomiju - Medicinskog Fakulteta u Rijeci. Program Donacije Tijela. https://anatomedri.com/donacije-tijela-za-medicinsku-edukaciju/ [Accessed July 15, 2025].

20 Bundesinstitut für öffentliche Gesundheit. Gesetz zur Stärkung der Entscheidungsbereitschaft bei der Organspende (2022). https://www.organspende-info.de/gesetzliche-grundlagen/gesetz-staerkung-entscheidungsbereitschaft/ [Accessed January 13, 2026].

21 German Organ Procurement Organization. https://www.dso.de/homepage-english [Accessed October 8, 2024].

22 Charité Universitätsmedizin Berlin. Anatomische Körperspende für die Charité Berlin. https://anatomie.charite.de/fuer_koerperspender [Accessed November 11, 2025].

23 European Federation for Organ Donation. National Laws on Organ Donation. https://www.efod.eu/wp-content/uploads/2021/11/Legislazione-Ungheria-ENG.pdf [Accessed July 23, 2025].

24 Organ Coordination Office. Welcome. https://www.ovsz.hu/en/organ-coordination-office/welcome [Accessed July 23, 2025].

25 Anatómiai - Szövet - és Fejlődéstani Intézet - Semmelweis Egyetem - Általános Orvostudományi Kar. HOLTTEST FELAJÁNLÁSA OKTATÁSRA. https://semmelweis.hu/anatomia/tetemfelajanlas-aktualis/ [Accessed July 24, 2025].

26 SanteSecu. Luxembourg-Transplant und Eurotransplant (2024). https://santesecu.public.lu/en/espace-citoyen/departement-sante/prevention/don-organes/luxembourg-transplant-et-eurotransplant.html [Accessed October 2, 2025].

27 Government of the Netherlands. Organ and tissue donation. https://www.government.nl/topics/organ-tissue-donation/rules-organ-tissue-donation [Accessed October 13, 2025].

28 Rijksoverheid. Contactgegevens andere (overheids)organisaties. https://www.rijksoverheid.nl/contact/contactgids/donorregister#:∼:text=Het%20Donorregister%20registreert%20of%20mensen,arts%20het%20Donorregister%20wil%20raadplegen [Accessed October 15, 2025].

29 Erasmus MC. Body donation. https://www.erasmusmc.nl/en/patient-care/body-donation [Accessed October 15, 2025].

30 Slovenija-transplant. About us. https://www.slovenija-transplant.si/en/about-us/ [Accessed November 2, 2025].

31 Inštitut za anatomijo - Univerza v Ljubljani. Izobraževanje na darovanih telesih. https://www.mf.uni-lj.si/ian/izobrazevanje-na-darovanih-telesih?q=%2Fian%2Fizobrazevanje-na-darovanih-telesih [Accessed November 3, 2025].

32 34 Lives. About. https://34lives.com [Accessed February 3, 2025].
